# 488. Comparison of Demographics and Clinical Characteristics of Multisystem Inflammatory Syndrome in Children and Kawasaki Disease

**DOI:** 10.1093/ofid/ofab466.687

**Published:** 2021-12-04

**Authors:** Rana Talj, Ahmad Yanis, Danielle A Rankin, Joseph R Starnes, Lauren S Starnes, Daniel E Clark, David Parra, Anna E Patrick, Sophie E Katz, Natasha B Halasa, Natasha B Halasa

**Affiliations:** 1 Vanderbilt University Medical Center, Nashville, Tennessee; 2 Vanderbilt University Medical Center; Division of Pediatric Infectious Diseases, Nashville, TN; 3 VUMC, Nashville, Tennessee

## Abstract

**Background:**

Multisystem inflammatory syndrome in children (MIS-C) is an illness associated with recent SARS-CoV-2 infection or exposure. Kawasaki disease (KD), a vasculitis with an unknown etiology, has overlapping clinical presentation with MIS-C, making it difficult to clinicians for distinguish between them. Therefore, we aimed to compare demographic, laboratory, and clinical characteristics between MIS-C and KD in hospitalized children in Nashville, TN.

**Methods:**

We conducted a single-center retrospective chart review for hospitalized children under 18 years who met American Heart Association criteria for KD and were treated with intravenous immunoglobulin from May 2000 to December 2019, and children meeting the CDC criteria for MIS-C from July 2020 to May 2021. Data abstraction for patients’ demographics, clinical presentation, laboratory values and imaging results was performed. Pearson’s chi-squared test for categorical variables and Wilcoxon rank sum test for continuous variables, with alpha=5%, were used to compare groups.

**Results:**

A total of 603 KD and 52 MIS-C hospitalized patients were included. Children with MIS-C were older than those with KD. A higher frequency of male sex was noted in both groups, with no significant differences in race and ethnicity (Table). MIS-C children frequently presented with symptoms similar to KD (63.5% rash, 55.8% conjunctivitis, 28.9% mucous membrane changes); however, only one MIS-C patient met criteria for complete KD (Figure). Both MIS-C and KD children presented with elevated CRP and ESR, but the median value of CRP in MIS-C children was significantly higher (Table). In addition, white cell count was lower in MIS-C children, which is primarily driven by the lower absolute lymphocyte count in this group (0.9 vs 2.7, p< 0.001), and echocardiography was more likely to be abnormal at presentation compared to KD (Table).

Table. Comparison of Sociodemographic, Clinical, and Laboratory Characteristics among Children with Kawasaki Disease and Multisystem Inflammatory Syndrome in Nashville

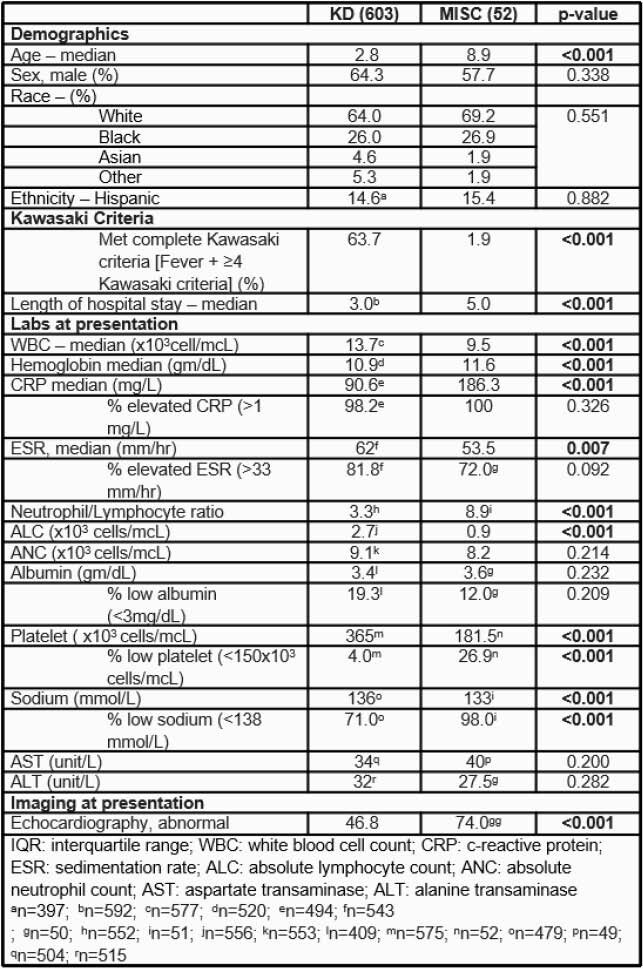

Figure. Comparison of Kawasaki Criteria Between Children with Multisystem Inflammatory Syndrome and Kawasaki Disease

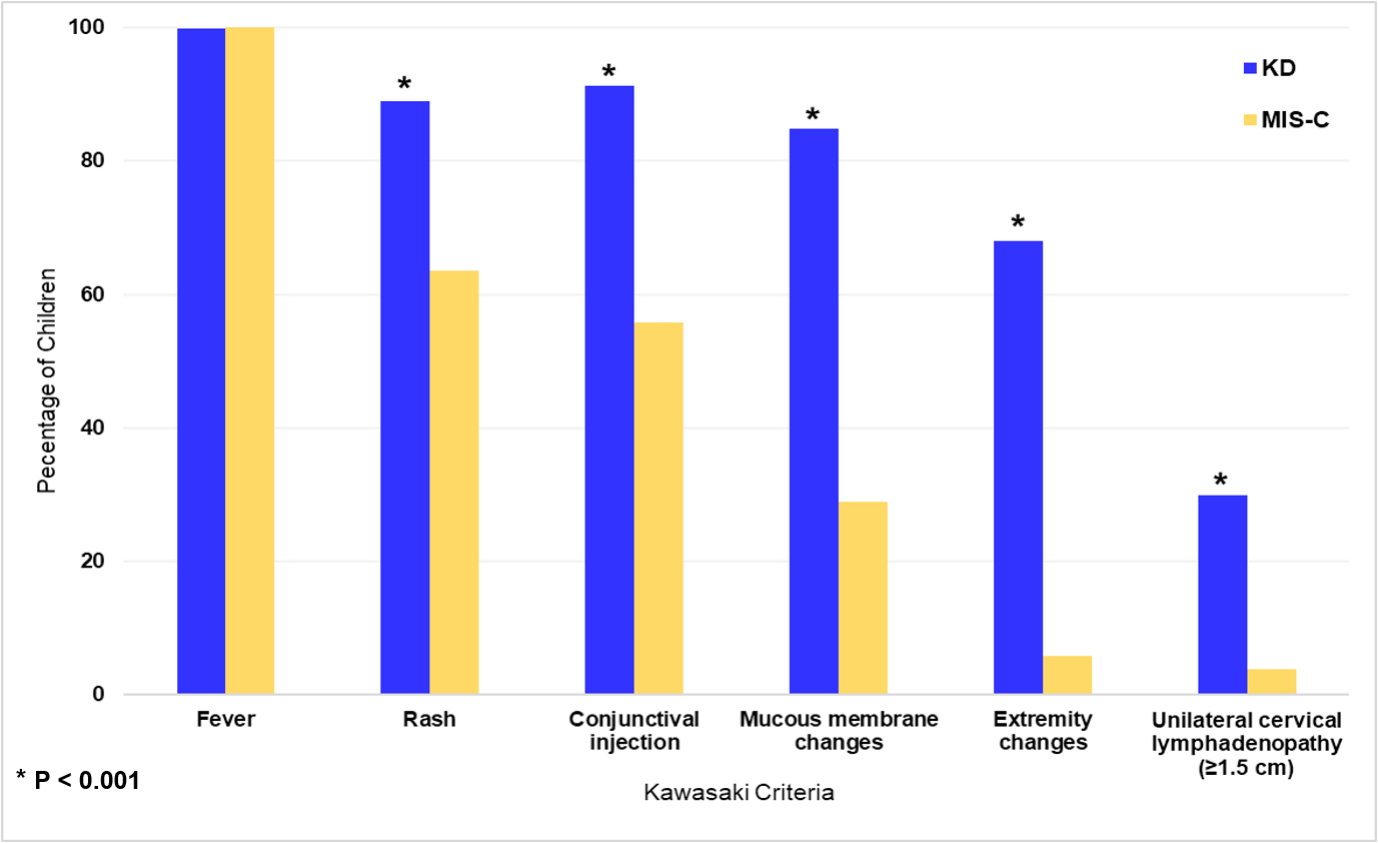

**Conclusion:**

MIS-C and KD present similarly in children; however, age, laboratory and echocardiography findings can help differentiate between them. Different laboratory values suggest different pathophysiology and inflammatory mediators behind these two illnesses, warranting further research.

**Disclosures:**

**Natasha B. Halasa, MD, MPH**, **Genentech** (Other Financial or Material Support, I receive an honorarium for lectures - it’s a education grant, supported by genetech)**Quidel** (Grant/Research Support, Other Financial or Material Support, Donation of supplies/kits)**Sanofi** (Grant/Research Support, Other Financial or Material Support, HAI/NAI testing) **Natasha B. Halasa, MD, MPH**, Genentech (Individual(s) Involved: Self): I receive an honorarium for lectures - it’s a education grant, supported by genetech, Other Financial or Material Support, Other Financial or Material Support; Sanofi (Individual(s) Involved: Self): Grant/Research Support, Research Grant or Support

